# Supplementary material for: Discovery of a new class of integrin antibodies for fibrosis
Source: Sci Rep. 2021 Jan 22;11:2118. doi: 10.1038/s41598-021-81253-0 (PMC7822819; doi:10.1038/s41598-021-81253-0)
Supplement: Supplementary file 1 — Supplementary Information. [file 41598_2021_81253_MOESM1_ESM.pdf]

Supplemental Information

Title: Discovery of A New Class of Integrin Antibodies for Fibrosis

Authors: Ji Zhang[1,]*, Tao Wang[2], Ashmita Saigal[1], Josephine Johnson[3], Jennifer Morrisson[2], Sahba Tabrizifard[2], Scott A. Hollingsworth[4], Michael J. Eddins[4], Wenxian Mao[3], Kim O'Neill[5], Margarita Garcia-Calvo[5], Ester Carballo-Jane[3], DingGang Liu[6], Taewon Ham[6], Qiong Zhou[6], Weifeng Dong[6], Hsien-Wei Meng[2], Jacqueline Hicks[7], Tian-Quan Cai[8], Taro Akiyama[1], Shirly Pinto[1], Alan C. Cheng[4], Thomas Greshock[7], John C. Marquis[2], Zhao Ren[3], Saswata Talukdar[1], Hussam Hisham Shaheen[2], and Masahisa Handa[2,]*

Affiliations: Departments of Cardiometabolic Diseases[1], Discovery Biologics[2], Quantitative Biosciences[3], Computational & Structural Chemistry[4], in vitro Pharmacology[5], SALAR[6], Discovery Chemistry[7], in vivo Pharmacology[8], MRL, Merck & Co., Inc., 2000 Galloping Hill Road, Kenilworth, NJ 07033, USA

One Sentence Summary: targeting integrin in lung fibrosis

Correspondence:

*Masahisa Handa, email: masahisa.handa@merck.com

*Ji Zhang, email: ji.zhang1@alumni.duke.edu

Supplemental Figures

Supplemental Fig. S1. Histology analysis of bleomycin-induced lung fibrosis model. A) Representative histological images of mouse lungs from each experimental group. Scale bar, 100 μM. Immunohistochemistry, IHC. Quantitative analysis of each lung lobe, B) Modified Ashcroft score, C) αSMA positive area, D) Area of Picosirus red (PSR) positive staining, and E) Percentage of CD68-positive cells. Mean±SEM, n=5. One-way ANOVA followed by Tukey's test, *p<0.05, **p<0.01, ***p<0.005 vs Saline group.

Supplemental Fig. S2. MK-0429 inhibits lung fibrosis in mouse bleomycin model. A) Body weight of each experimental group over the duration of time course. Mean±SEM, n=10. One-way ANOVA followed by Tukey's test, **p<0.01, ***p<0.001 vs Saline group; ##p<0.01, ###p<0.001 vs BLM-vehicle group. B) Percentage of body weight changes in each experimental group. Mean±SEM, n=10. One-way ANOVA followed by Tukey's test, **p<0.01, ***p<0.001 vs Saline group; ##p<0.01, ###p<0.001 vs BLM-vehicle group. C) TIMP1 concentration in BALFs. Mean±SEM, n=10. One-way ANOVA followed by Tukey's test, *p<0.05, **p<0.01, ***p<0.001 vs BLM-vehicle group.

Supplemental Fig. S3. The expression of αv integrins in CHOK1 stable lines. A) FACS of mouse αv and β1 expression in CHOK1-α5KO-mαvβ1 cells. Anti-αv (RMV7) antibody and anti-β1 (KMI6) antibody were used for detection. B) FACS of mouse αv and β3 expression in CHOK1-mαvβ3 cells. Anti-αv (RMV7) antibody and anti-β3 (HMβ3.1) antibody were used for detection. C) FACS of mouse αv and β5 expression in CHOK1-mαvβ5 cells. Anti-αv (RMV7) antibody and anti-β5 (P1F76) antibody were used for detection. D) FACS of mouse αvβ6 expression in CHOK1-mαvβ6 cells. Anti-αvβ6 (10D5) antibody was used for detection. E) FACS of mouse αv and β8 expression in CHOK1-mαvβ8 cells. Anti-αv (RMV7) antibody and anti-β8 (ADWA11) antibody were used for detection.

Supplemental Fig. S4. Integrin antibodies with strong blocking activities against both human and mouse αv integrins. A) A) Titration of Ab-29, Ab-30, Ab-31, Ab-32, and Ab-33 for their binding to CHOK1-mouse αvβ1, αvβ6, and α5β1 stable cell lines in CELISA assays. B) Dose-dependent inhibition of mouse integrin-ligand binding by MK-0429 in AlphaLISA assay panel. C) Octet Red binding parameter of Ab-29, Ab-30, Ab-31, Ab-32, and Ab-33 to selected human and mouse integrins.

Supplemental Fig. S5. MK-0429 inhibits integrin-mediated cell adhesion. A) The effect of MK-0429 on the adhesion of CHOK1 parental, CHOK1-α5KO-mαvβ1, CHOK1-mαvβ3, and CHOK1-mαvβ5 cells to fibronectin or vitronectin matrix.

Supplemental Fig. S6. Sally Sue simple western full-length blot images presented in Fig. 1. A-E) blot images presented in Fig. 1A. The expression of various integrins in human primary lung cell types upon TGFβ (5ng/ml for 24 hours) treatment. Following immunoprecipitation with an anti-αv antibody, the αvβ1, αvβ3, αvβ5, and αvβ6 heterodimers were detected by Sally Sue simple western analysis after using antibodies that recognize each individual β-subunit. Normal human lung fibroblast, NHLF; normal human bronchial epithelial cells, NHBE; small airway epithelial cells, SAEC; bronchial smooth muscle cells, BSMC; pulmonary artery smooth muscle cells, PASMC; pulmonary artery endothelial cells, PAEC. Integrin antibodies were reported in Supplemental Table S2. Exposure time: 4 seconds. F-N) blot images presented in Fig. 1C. Integrin expression and signaling in fibrotic lungs was determined by Sally Sue simple western analysis using antibodies that recognized the individual subunits. GAPDH level in total lung lysates was used as a loading control. Integrin antibodies were reported in Supplemental Table S2. Exposure time: 4 seconds.

Supplemental Table S1. Recombinant human and mouse integrin expressing constructs.

Supplemental Table S2. Integrin antibodies used for FACS and Sally Sue simple western.

Supplemental Table S3. AlphaLISA assay condition and reagents.

Supplemental Fig S1

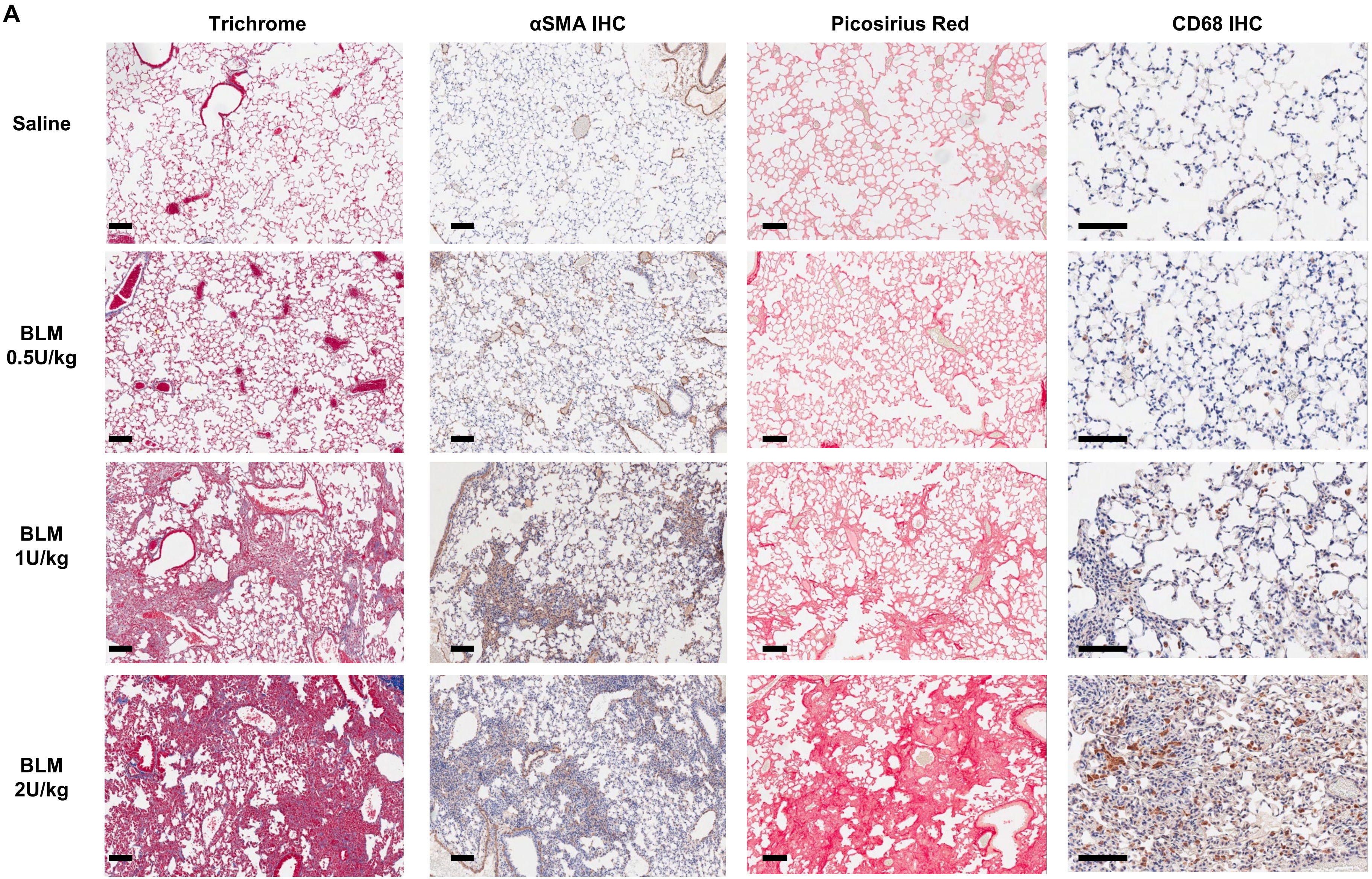

Supplemental Fig S1

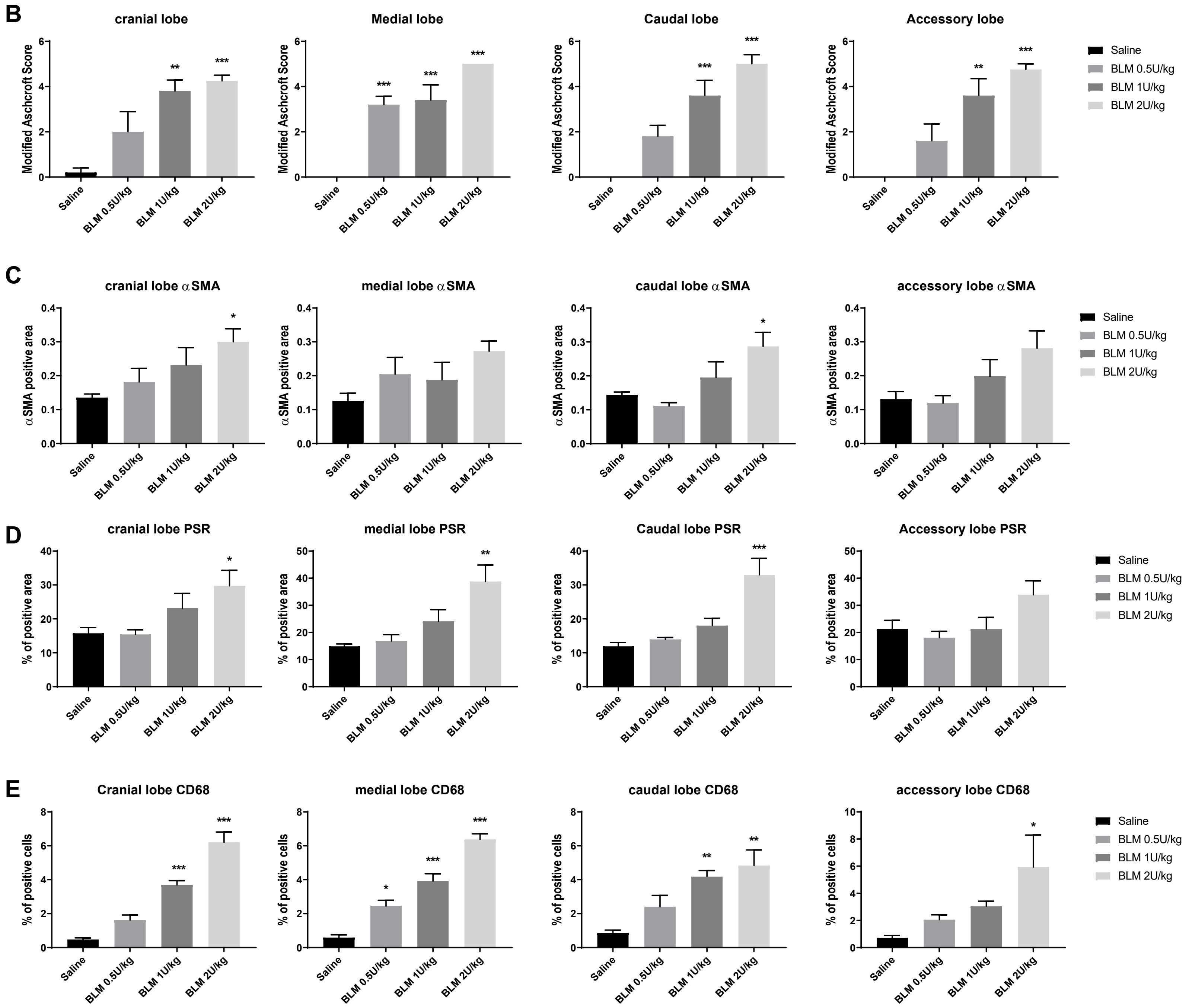

Supplemental Fig S2

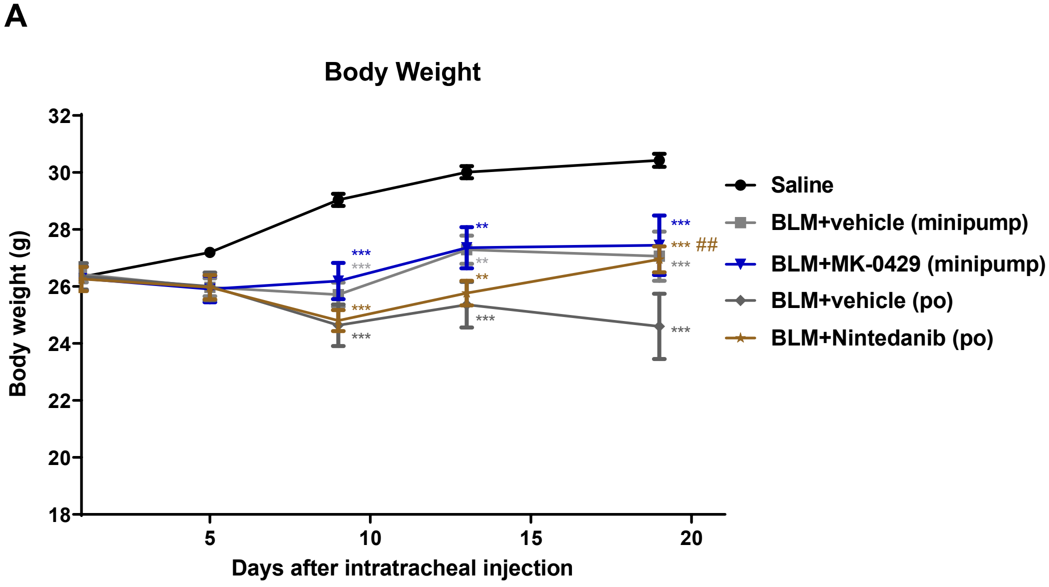

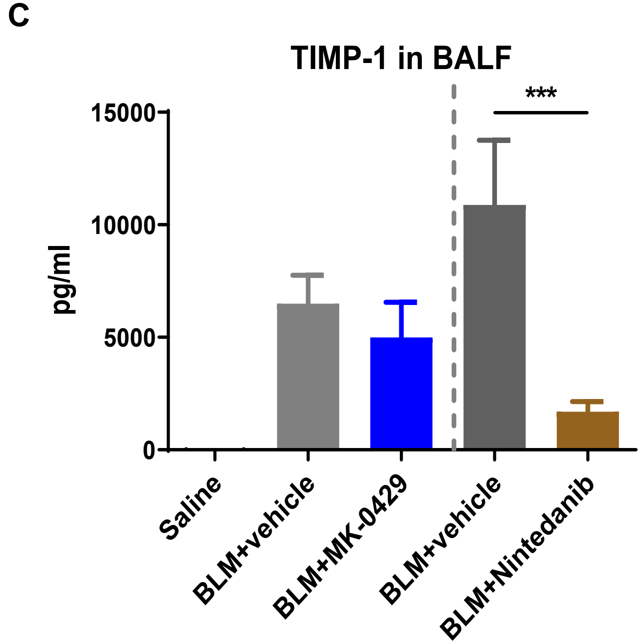

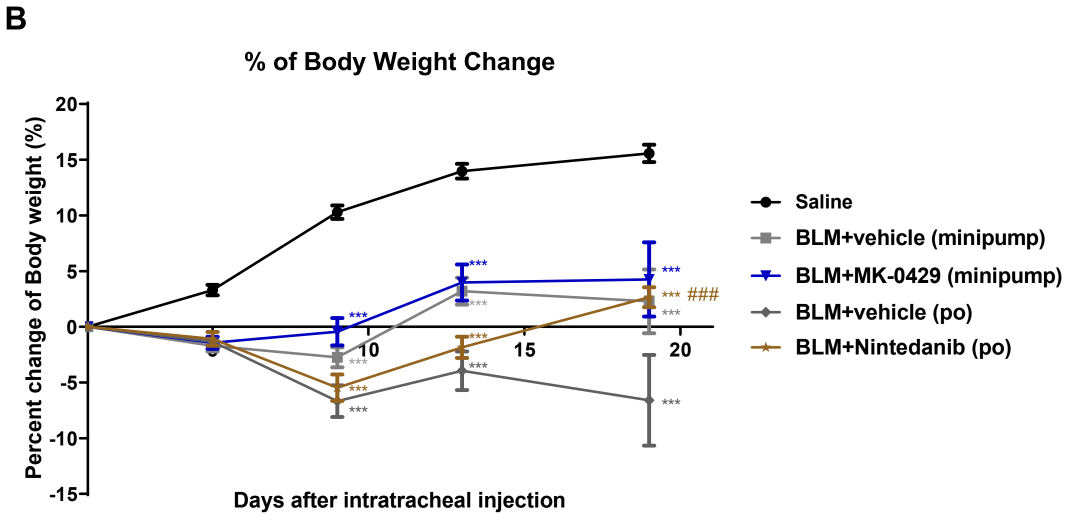

Supplemental Fig S3

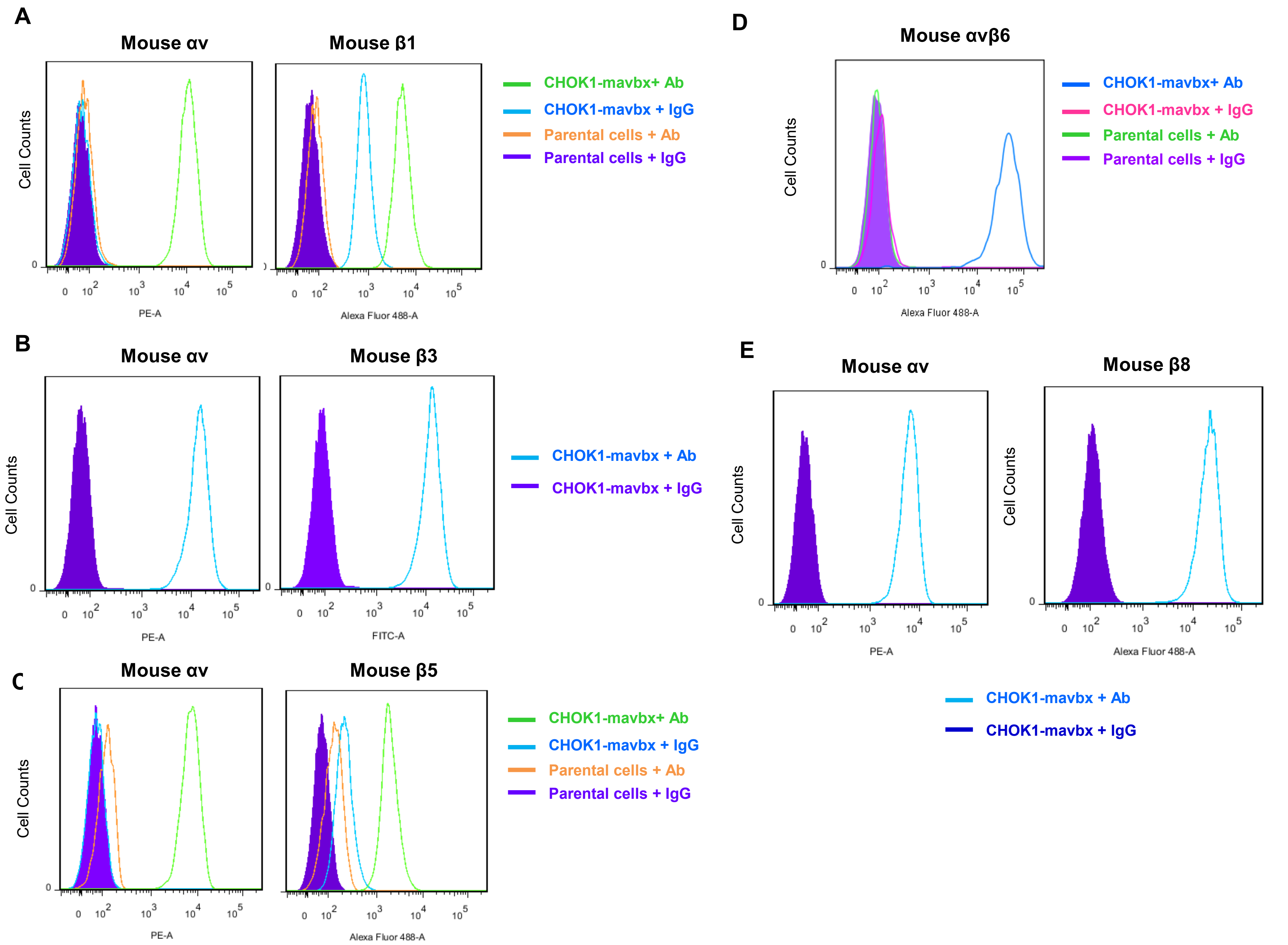

Supplemental Fig S4

A

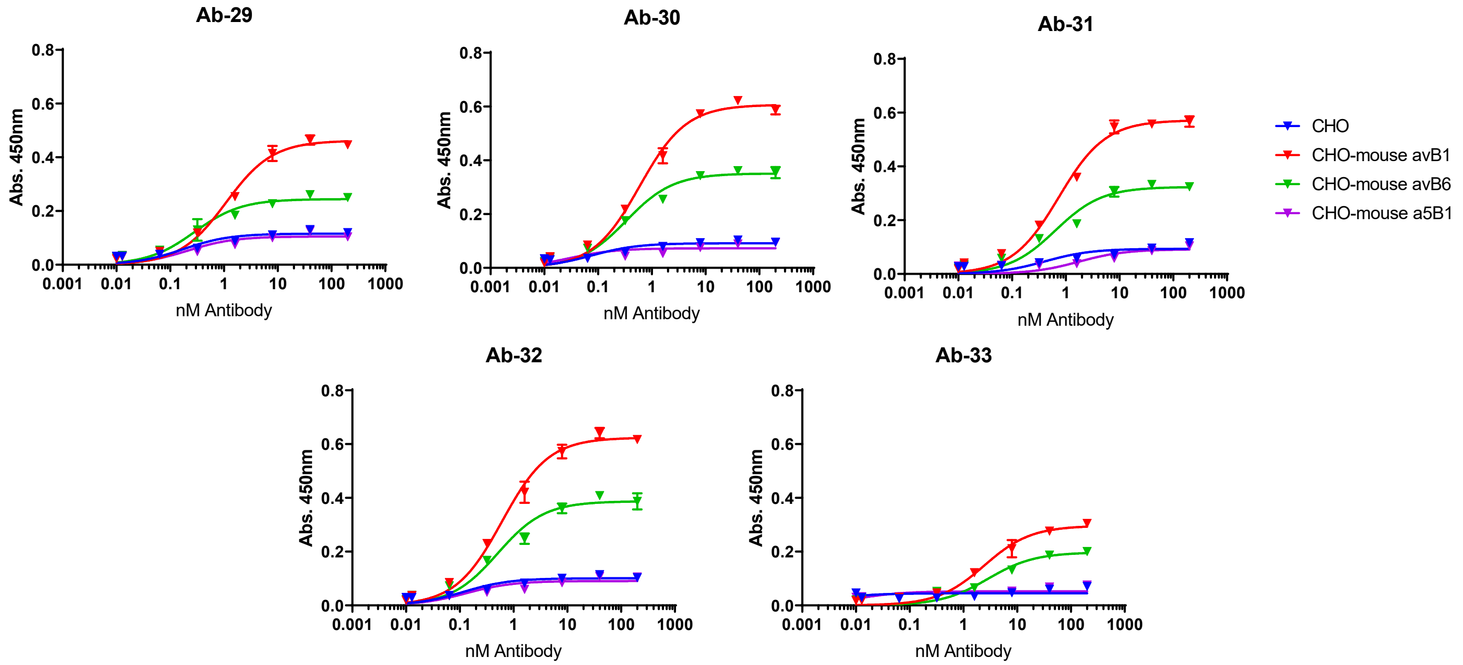

B

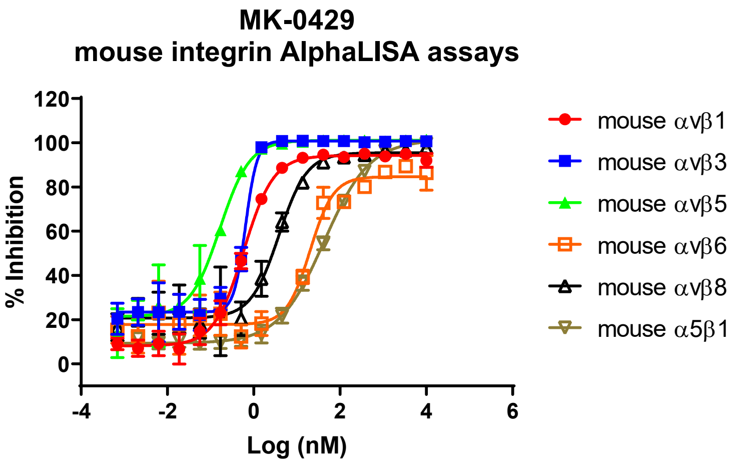

C

Clone Name	hαVβ1 KD (M)	hαVβ3 KD (M)	mαVβ1 KD (M)	mαVβ6 KD (M)
Ab-29	2.70E-09	6.58E-09	4.21E-09	4.11E-09
Ab-30	6.75E-09	5.29E-09	3.5E-08	3.03E-09
Ab-31	1.40E-08	6.47E-09	4.84E-08	2.9E-09
Ab-32	7.40E-09	6.14E-09	2.59E-08	2.59E-09
Ab-33	1.27E-08	5.07E-09	3.77E-08	2.45E-09

Supplemental Fig S5

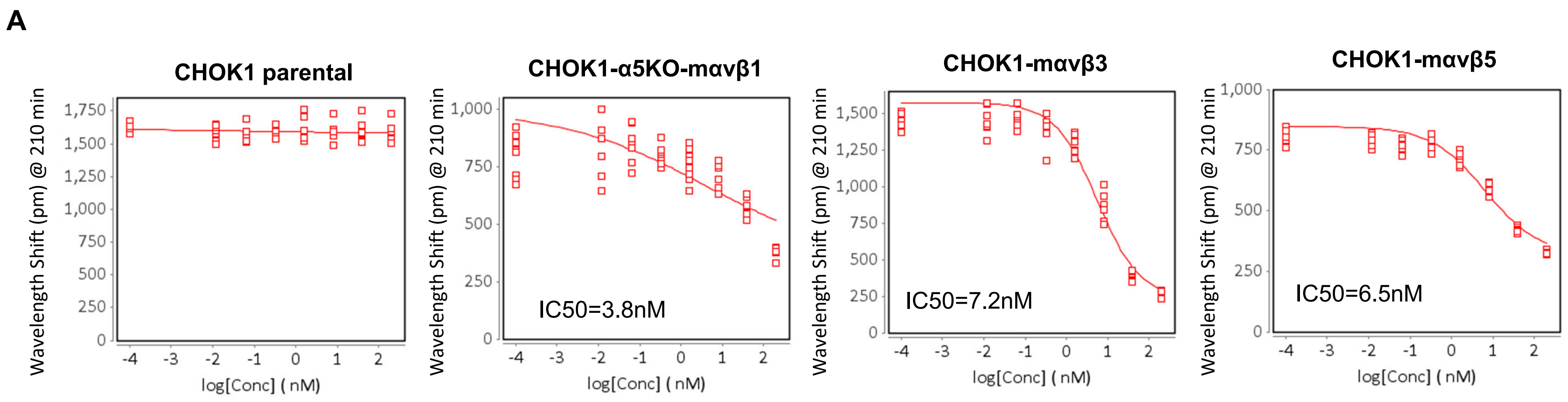

Supplemental Figure S6

A

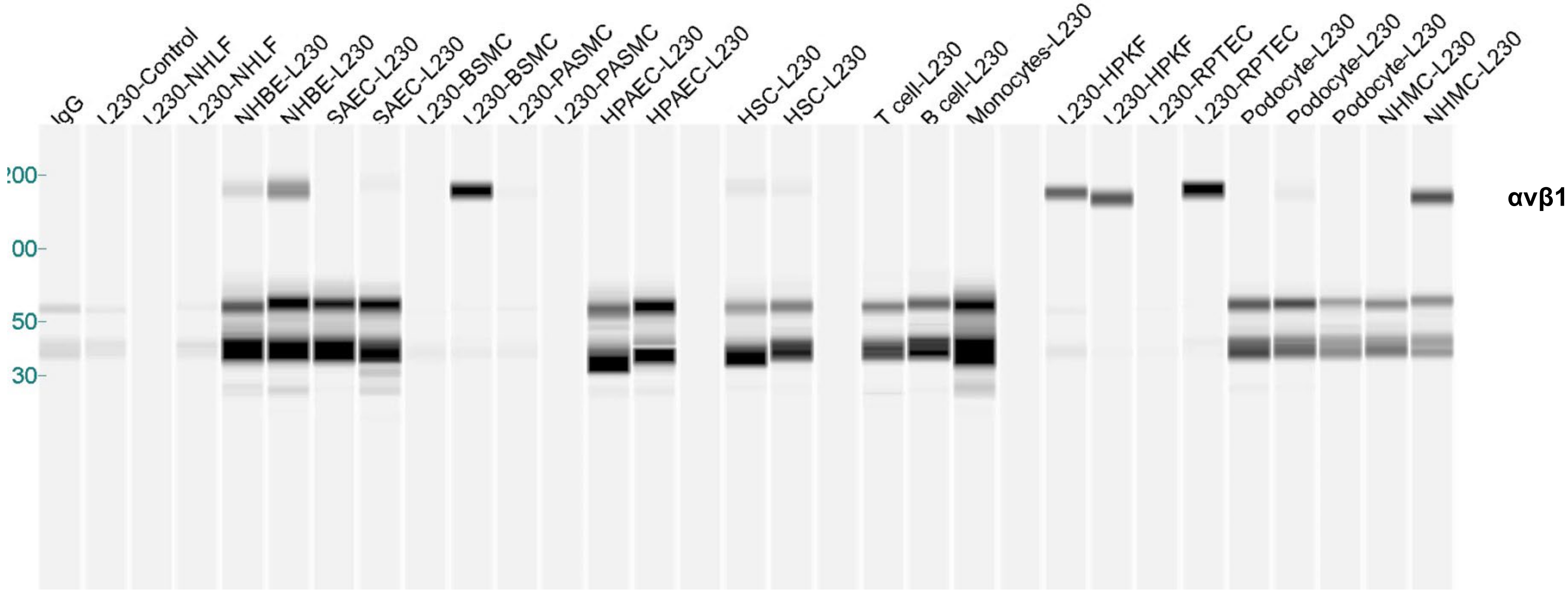

αvβ1

B

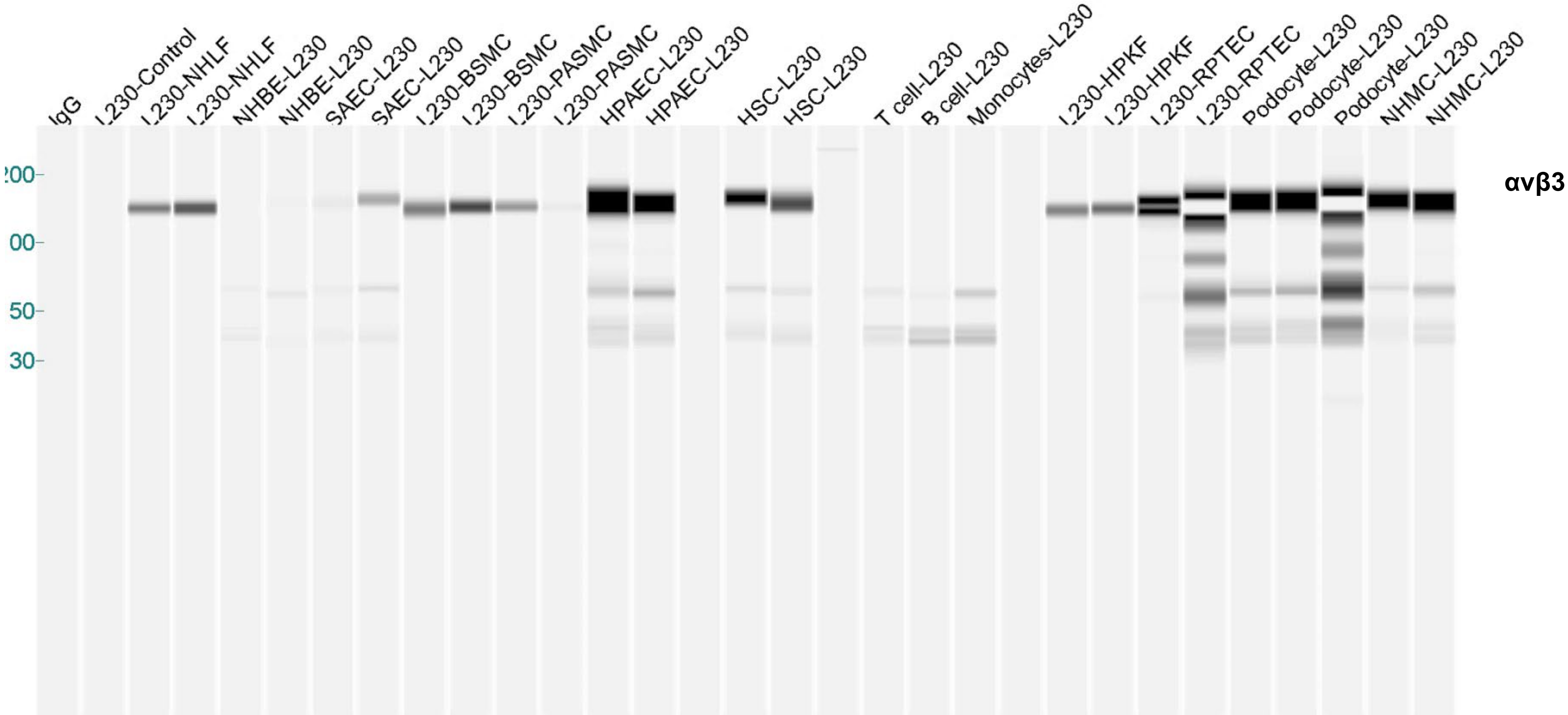

αvβ3

Supplemental Figure S6

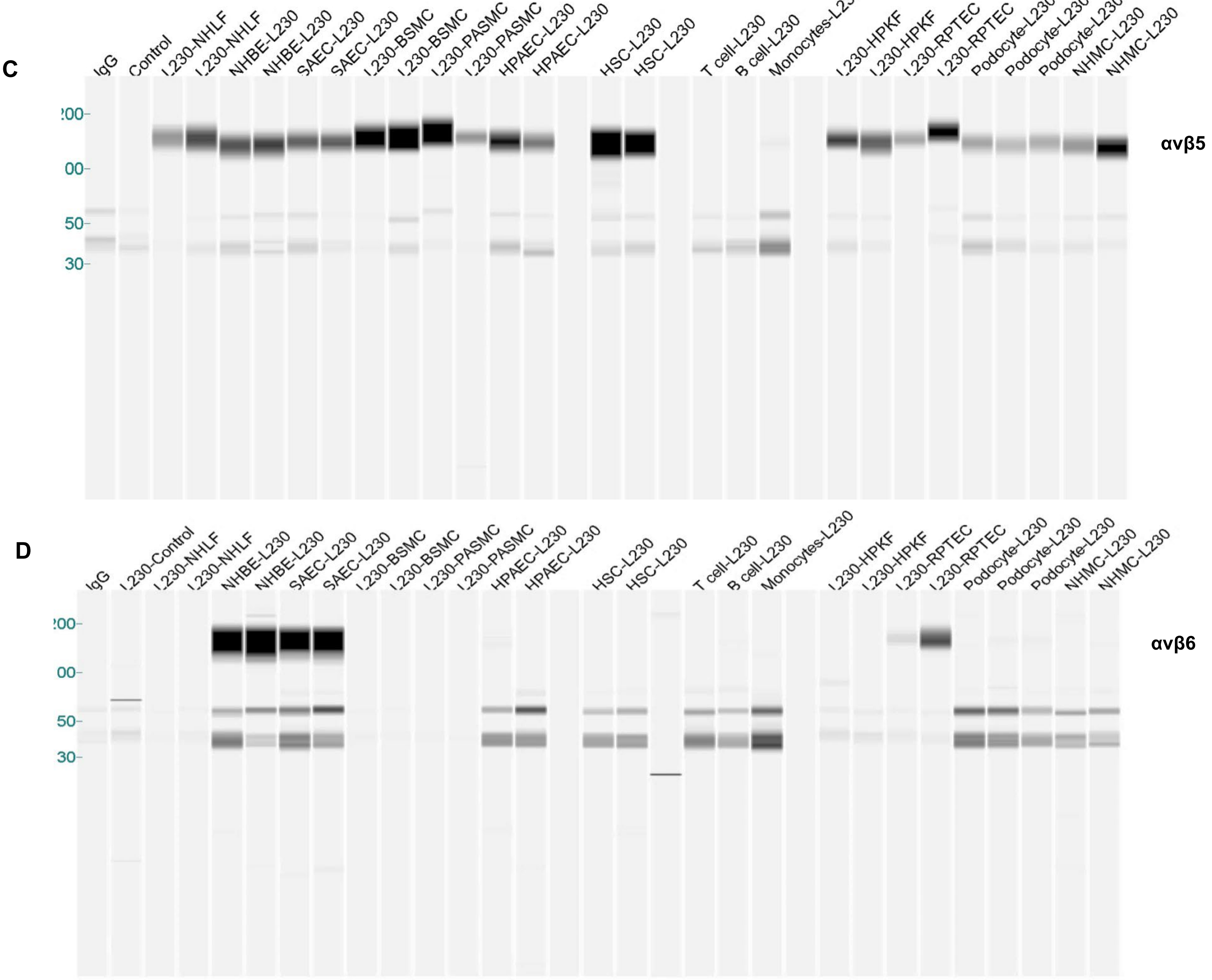

Supplemental Figure S6

E

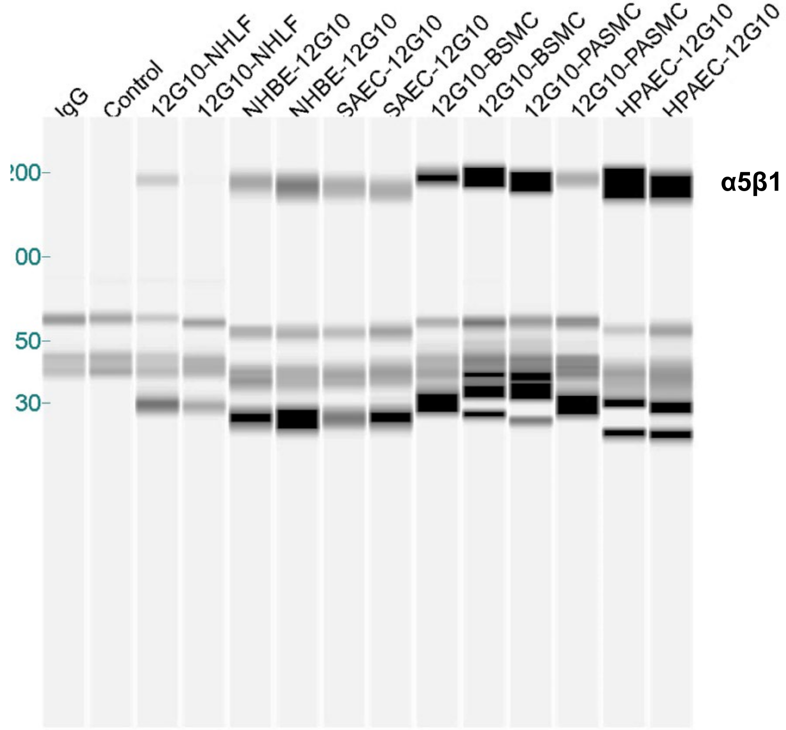

F

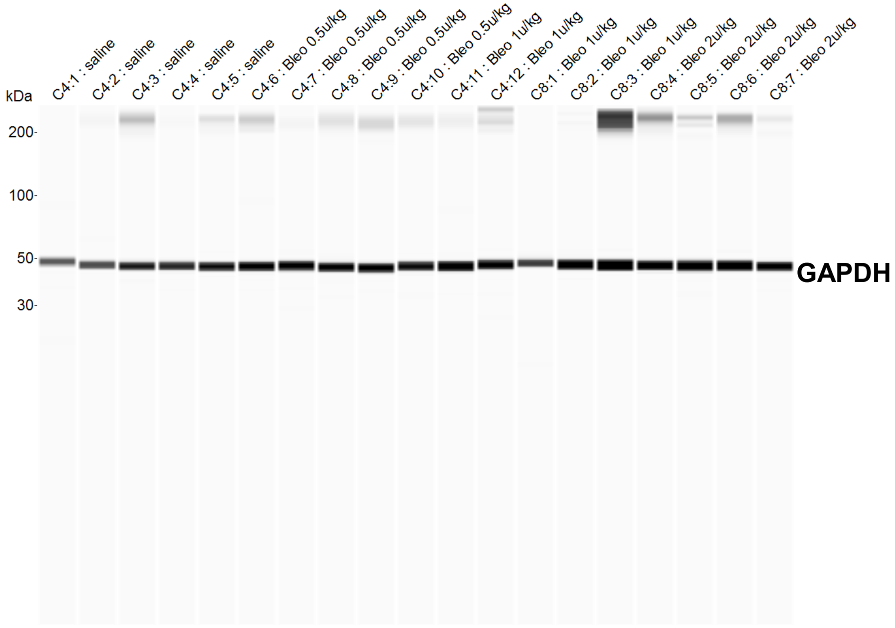

Supplemental Figure S6

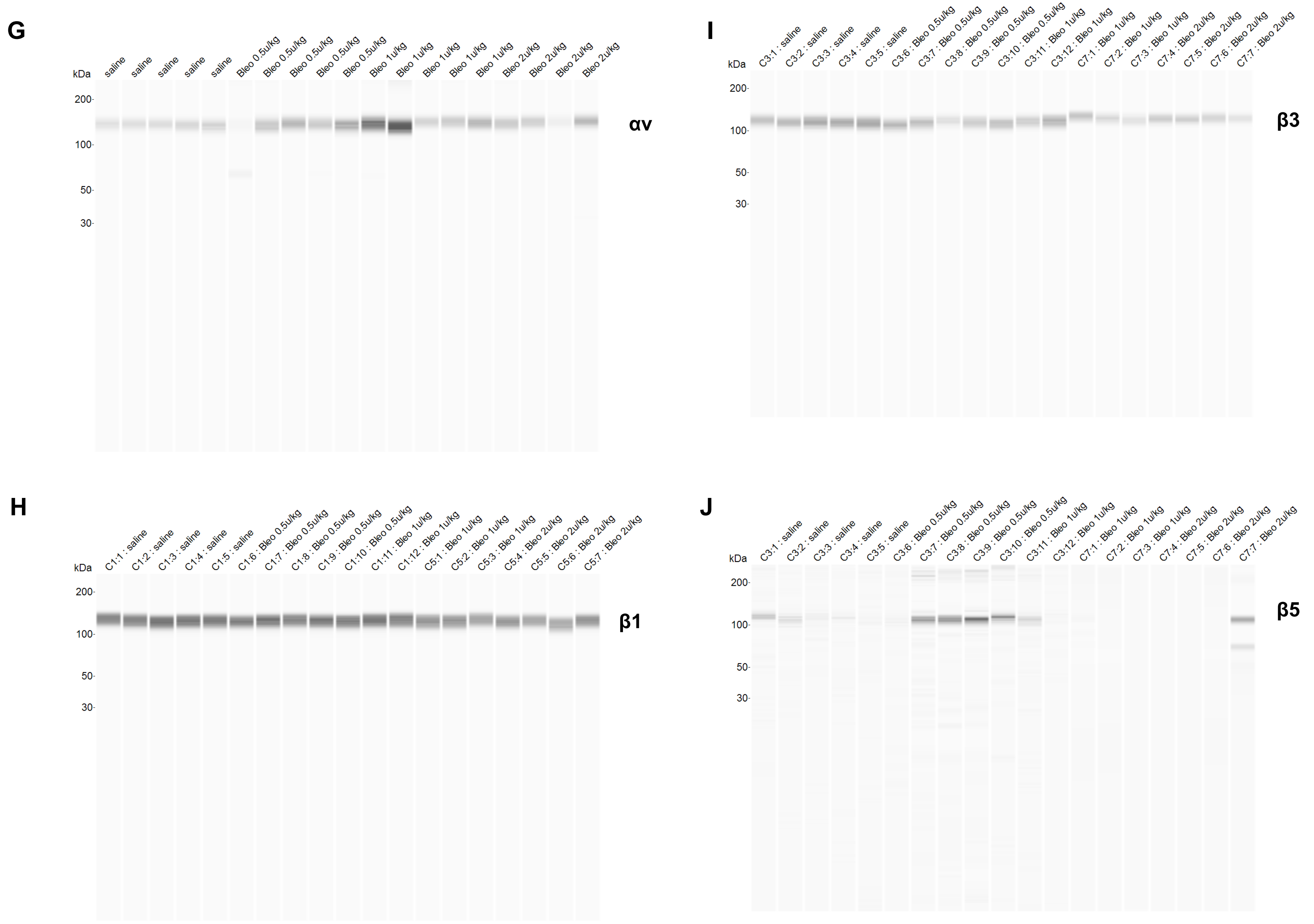

Supplemental Figure S6

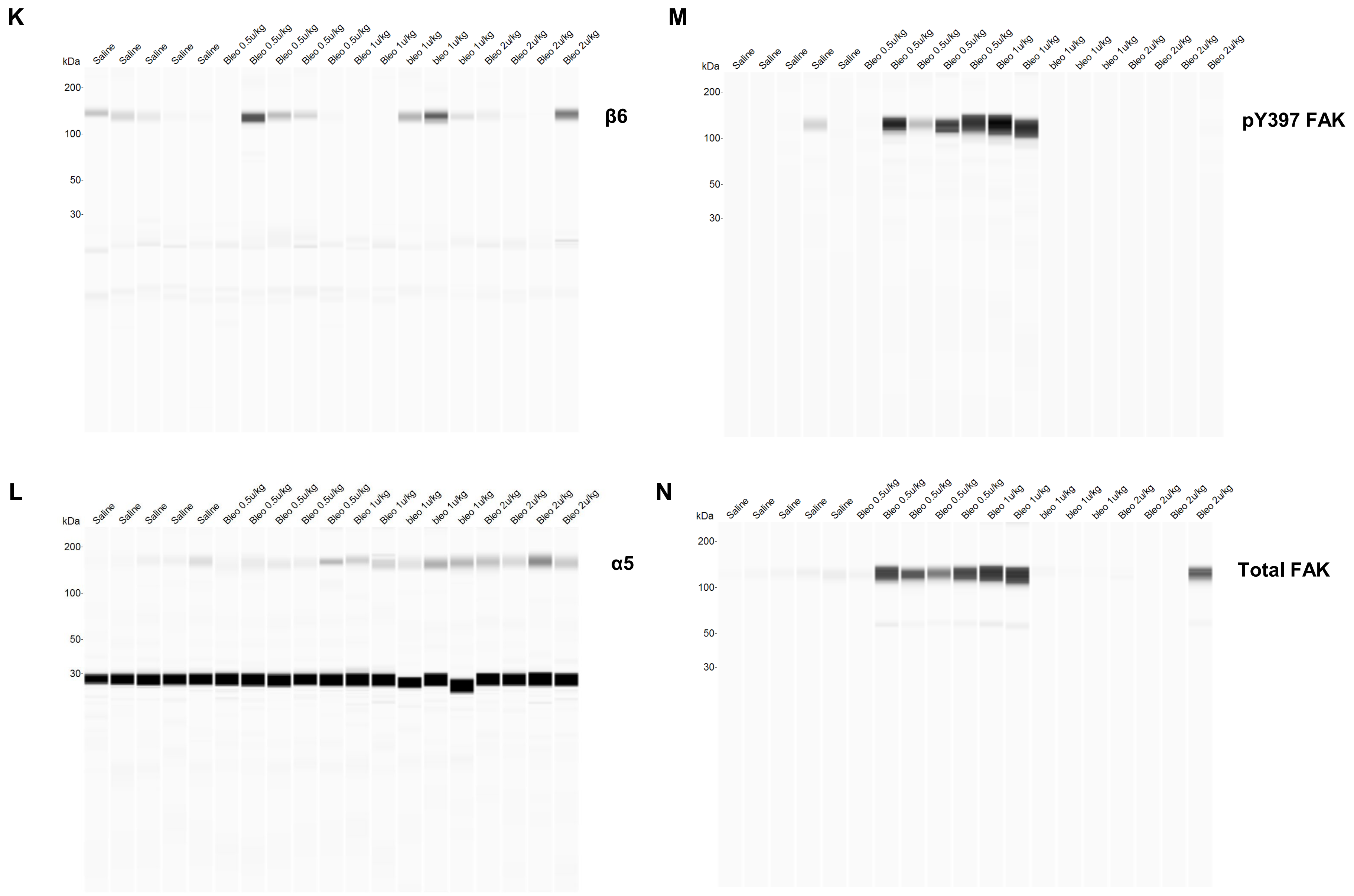

Supplemental Table S1. recombinant human and mouse integrin expressing constructs

Integrin subunit	N-term	C-term
hαV	1	992
hα5	1	995
hβ1	1	728
hβ3	1	718
hβ5	1	719
hβ6	1	709
hβ8	1	684
mαV	1	988
mα5	1	999
mβ1	1	728
mβ3	1	717
mβ5	1	719
mβ6	1	706
mβ8	1	679

Supplemental Table S2. integrin antibodies used for FACS and Sally Sue simple western.

Integrins	Clone	Company	Catalog #	Isotype	Reactivity
α5	HMα5-1	BD	553350	hamster IgG1	mouse
α5	EPR7854	Abcam	150361	rabbit IgG	human, mouse
α5	PB1	DSHB		mouse IgG1	hamster
αv	RMV-7	BD	552299	rat IgG1	mouse
αv	P2W7	R&D Systems	MAB1219	mouse IgG1	human
β1	KMI6	BD	558741	rat IgG2a	mouse
β1	P5D2	R&D Systems	MAB17781	mouse IgG1	human
β1	7E2 (DSHB)	DSHB		mouse IgG1	hamster
β3	HMβ3.1	BD	553347	hamster IgG1	
αvβ3	27.1(VNR-1)	Abcam	ab78289	mouse IgG1	human
β5	P1F6	Millipore	MAB1961	mouse IgG1	mouse
αvβ5	P5H9	R&D Systems	MAB2528	mouse IgG1	human
αvβ6	10D5	BD	566922	mouse IgG2a	human, mouse
β8	ADWA11	-	-	mouse IgG1	human, mouse
β8	416922	LSBio	LS-C70803	mouse IgG2b	human

Supplemental Table S3. integrin AlphaLISA assay reagents and conditions

Human Alphalisa Integrin Assays: Final Conditions

Integrin EC80 (batch dependent)			Ligand			Acceptor Bead Solution						Donor		
						antibody			AlphaLisa Acceptor					
havb1	1.4	nM	human Fibronectin R&D Systems (1918-FN)	1.0	nM	n/a			α - Fibronectin Acceptor beads/ Perkin Elmer (CUSM03822000) See notes	10.0	ug/ml	Streptavidin Donor beads/ Perkin Elmer (6760002)	10.0	ug/ml
ha5b1	0.4	nM		0.5	nM									
havb3	3.0	nM	human Vitronectin-GST tagged EMD Millipore (08-126)	10.0	nM	n/a			α - GST Acceptor beads/ Perkin Elmer(AL110C)	15.0	ug/ml		15.0	ug/ml
havb5	2.5	nM		1.0	nM									
havb6	0.1	nM	human LAP R&D system (246-LP)	0.5	nM	α-Human LAP (goat pAb) R&D system (AF-246-NA)	3.0	nM	α - goat IgG Acceptor beads/ Perkin Elmer (AL107C)	15.0	ug/ml		15.0	ug/ml
havb8	1.0	nM		3.0	nM		3.0	nM						

Mouse Alphalisa Integrin Assays: Final Conditions

Integrin EC80 (batch dependent)			Ligand			Acceptor Bead Solution						Donor		
						antibody			AlphaLisa Acceptor					
maVb1	4.3	nM	mouse fibronectin Abcam (ab92784)	1.0	nM	α - Fibronectin (Rabbit pAb) Abcam(ab2413)	5.0	nM	α-rabbit IgG Acceptor/ Perkin Elmer (AL104C)	15	ug/ml	Streptavidin Donor beads/ Perkin Elmer (6760002)	15	ug/ml
ma5b1	1.0	nM		2.5	nM									
maVb3	3.0	nM	mouse Vitronectin Abcam (ab92727)	5.0	nM	α - Vitronectin (Rabbit pAb) Abcam (ab140016)	2.0	nM						
maVb5	0.6	nM		2.5	nM									
maVb6	1.4	nM	mouse LAP-6His Chempartner	2.5	nM	n/a			α-6xHIS/ Perkin Elmer (AL128)	15	ug/ml		15	ug/ml
maVb8	1.0	nM		2.5	nM									
